# Wandering Lymphoma: A Case of Wandering Spleen Secondary to Splenic Marginal Zone Lymphoma

**DOI:** 10.1155/crh/8172177

**Published:** 2026-05-27

**Authors:** Joseph Brandon Parker, Claire Wilson, Ricardo Parrondo, Muhamad Alhaj Moustafa

**Affiliations:** ^1^ Department of Internal Medicine, Mayo Clinic Florida, 4500 San Pablo Road, Jacksonville, Florida, 32224, USA, mayoclinic.org; ^2^ Department of Hematology and Oncology, Mayo Clinic Florida, 4500 San Pablo Road, Jacksonville, Florida, 32224, USA, mayoclinic.org

**Keywords:** lymphoma, splenectomy, splenic marginal zone lymphoma, splenomegaly, wandering spleen

## Abstract

**Background:**

Splenic marginal zone lymphoma (SMZL) is a rare subtype of non‐Hodgkin lymphoma. It often follows an indolent course presenting with splenomegaly. Treatment varies from clinically monitoring to splenectomy, chemotherapy, and rituximab treatments. Wandering spleen is an even rarer condition with less than 500 cases reported in the literature, often resulting from laxity in the suspensory ligaments supporting the spleen. Wandering spleen most commonly affects women of childbearing age and children. Treatment for this can be either nonsurgical or surgical with splenopexy or splenectomy.

**Case Presentation:**

A 75‐year‐old female initially presented from Mexico for evaluation of splenomegaly and abdominal distension. Computer tomography (CT) scan of the abdomen and pelvis showed a pelvic mass, and labs showed leukocytosis with lymphocytic predominance. The pelvic mass was later confirmed as a wandering spleen. Flow cytometry on peripheral blood was diagnostic for SMZL. The patient was diagnosed with wandering spleen secondary to SMZL and treated with splenectomy.

**Conclusion:**

Wandering spleen is very rare and is typically associated with pregnancy, splenomegaly, or inborn errors of the ligaments supporting the spleen. This is only the fifth case reported in the literature of wandering spleen secondary to a lymphoma and the first reported with SMZL. This case offers a simultaneous presentation of two very rare conditions presenting in an atypical fashion. SMZL commonly presents in this age group; however, wandering spleen is rarely seen at this age. Additionally, this patient’s first‐line treatment for her SMZL was nontraditional with only a splenectomy with no rituximab administered due to the risk of torsion and infarction of the wandering spleen.

## 1. Background

Lymphomas are malignancies of the immune system and are divided into Hodgkin’s lymphoma (HL) and non‐Hodgkin’s lymphoma (NHL) [1]. Lymphoma has a prevalence in the United States of 879,242 in the year 2024 [2]. Splenic marginal cell lymphoma (SMZL) is a rare subtype of NHL and represents less than 2% of all lymphoid neoplasms [3]. The median age at diagnosis is 69 years [3]. SMZL often presents with splenomegaly [3–5]. Patients could also present with anemia, thrombocytopenia, and leukocytosis [4, 6]. SMZL often follows an indolent course with a variable 10‐year survival probability ranging between 42% and 95% [4, 6]. Adverse clinical prognostic factors include large tumor burden and poor performance status [4]. Genetic factors that are associated with poor outcomes are the presence of p53 mutations, 7q deletion, NOTCH2 mutation, and the absence of somatic mutation in IgVH genes [4].

Treatment for SMZL varies due to the rarity of this lymphoma [7]. In asymptomatic patients, occurring in approximately one‐third of patients with SMZL, clinical observation is the standard [8–11]. Treatment should be initiated only in the presence of symptoms including symptomatic splenomegaly, cytopenia, or systemic symptoms [10]. Treatment options for symptomatic patients include splenectomy, rituximab, chemotherapy, or rituximab and chemotherapy combinations [12]. Rituximab alone or in combination with chemotherapy is the first line of therapy for SMZL, showing better outcomes to those treated with chemotherapy only [13]. One study showed similar survival outcomes in patients who underwent rituximab treatment versus splenectomy [14]. Specifically, this study cited that rituximab monotherapy is a valuable alternative to patients over 65 years of age [14]. Overall, splenectomy is appropriate for those fit for surgery, and they may not require further treatment [13, 15, 16]. However, rituximab with or without splenectomy should be considered for the first‐line treatment of SMZL [13]. Herein, we describe a unique case of SMZL that presented with wandering spleen and was treated with splenectomy only.

## 2. Case Presentation

**FIGURE 1:**
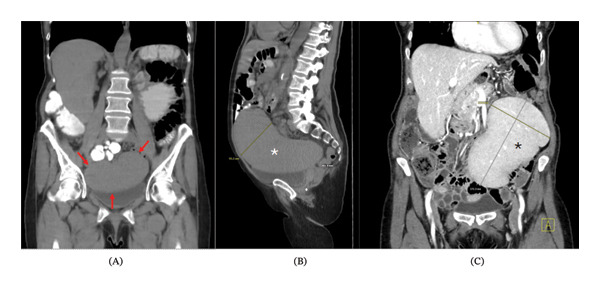
Abdominal computed tomography (CT) suggestive of a pelvic tumor measuring 20 × 9.5 × 13 cm (Panel (A) with red arrows, Panel (B) with white asterix, Panel (C) with the black asterix). Panels (A) and (B) are from the CT abdomen/pelvis performed in April 2024, and Panel (C) is from the CT abdomen/pelvis performed in June 2024.

A 75‐year‐old female presented for evaluation of abdominal fullness, distension, and early satiety. Six years prior to her presentation to our institution, she was misdiagnosed with chronic lymphocytic leukemia (CLL) due to her high lymphocyte count. Prior to arrival to our facility, she had a computed tomography (CT) scan of the abdomen and pelvis that showed a pelvic mass. She presented to our institution for a second opinion regarding her CLL. Our laboratory workup demonstrated leukocytosis (WBC 29.2 × 10^9^/L, hemoglobin 12.7 g/dL, and platelet count of 152 × 10^9^/L) with the differential showing lymphocytic predominance (neutrophils 9.7%, lymphocytes 76.3%, eosinophils 2.4%, monocytes 1.6%, and basophils 0%). Her physical exam was remarkable for abdominal distension. She had a CT scan of the abdomen and pelvis which showed a pelvic mass (18.5 × 9.1 × 8 cm in the anterior–posterior cephalocaudal direction) that was then discerned to be an enlarged spleen in a wandering position. Images of the two CT abdomen/pelvis scans can be seen in Figure 1. Subsequently, flow cytometry on the peripheral blood revealed a monoclonal B‐cell population that is positive for CD19 and CD20 and negative for CD5, CD10, and CD23, consistent with SMZL. Due to the risk of torsion and infarct, an open splenectomy was subsequently performed. Biopsies were collected from the spleen and confirmed the diagnosis of SMZL.

## 3. Discussion and Conclusion

Wandering spleen is a condition in which a spleen fails to fixate in its normal location in the left upper quadrant of the abdomen [17]. It is a rare condition that occurs when there is lack of, laxity, or weakening of the spleen’s suspensory ligaments [18]. It often occurs in the setting of splenomegaly or pregnancy but can occur from inborn error, trauma, and connective tissue disorders [19]. Wandering spleen commonly affects those between the ages 20 and 40 and is more common in women but can also affect children less than 1 year of age [19]. Symptoms can include abdominal pain or fullness, abdominal or pelvic distension, but often is an incidental finding [20]. Treatment for wandering spleen can be either nonsurgical or surgical. Nonsurgical is not the preferred treatment due to the high risk of complications in this population, with risk as high as 65%, and complications include torsion and infarction [18]. For surgical intervention, there are two options, splenopexy or splenectomy, depending on the case and viability of the spleen [21]. Additionally, surgical treatment of wandering spleen is typically not altered by the presence of a hematologic malignancy. Causes of wandering spleen documented in the literature can be seen in Table 1. Wandering spleen has been rarely documented with lymphoma, with only four cases reported in the literature [37–40].

**TABLE 1:** List of causes of wandering spleen cited in the literature with respective selected citations corresponding and approximate number of cases.

Cause of wandering spleen	Selected references	Approximate number of cases reported
Congenital anomalies	[22–25]	100+
Pregnancy (hormonal effects)	[26–29]	15+
Splenomegaly	[30, 31]	10+
Connective tissue disorders	[32, 33]	2
Trauma	[34]	8+
Diaphragmatic hernia	[35, 36]	5+
Lymphoma	[37–40]	4

In this patient’s case, her wandering spleen most likely resulted from her splenomegaly secondary to her SMZL with laxity in her ligaments supporting the spleen. In most cases of SMZL, single‐agent rituximab with or without splenectomy is the mainstay of treatment [13]. However, in this case, splenectomy was the primary treatment of choice due to the lymphoma being complicated by the wandering spleen to avoid risk of torsion or infarction. Additionally, no further treatment with chemotherapy or rituximab was performed due to the patient having no other symptoms. In summary, SMZL could rarely present with a wandering spleen, and due to high risk of torsion and infarction, splenectomy should be considered. In addition, wandering spleen should not be missed when evaluating a pelvic mass in the absence of normally located spleen due to high risk for bleeding if biopsies are done.

**Nomenclature**
HL: Hodgkin’s lymphoma
NHL: Non‐Hodgkin’s lymphoma
SMZL: Splenic marginal zone lymphoma
CLL: Chronic lymphocytic leukemia
CT: Computed tomography

## Author Contributions

Joseph Brandon Parker: writing–original draft, writing–review and editing, investigation, software, and methodology.

Claire Wilson: writing–review and editing, investigation, and software.

Ricardo Parrondo: writing–review and editing, investigation, visualization, and conceptualization.

Muhamad Alhaj Moustafa: supervision, writing–review and editing, investigation, visualization, and conceptualization.

## Funding

This article has no funding source.

## Ethics Statement

Consent was obtained from the patient for publication.

## Conflicts of Interest

The authors declare no conflicts of interest.

## Data Availability

The data that support the findings of this study are available on request from the corresponding author. The data are not publicly available due to privacy or ethical restrictions.
